# A single-cell and spatial atlas of plaque macrophage states in human atherosclerosis

**DOI:** 10.3389/fimmu.2026.1823101

**Published:** 2026-04-29

**Authors:** Yuanyuan Yang, Haiying Zhu

**Affiliations:** Department of Cell Biology, Naval Medical University (Second Military Medical University), Shanghai, China

**Keywords:** atherosclerosis, heterogeneity, macrophage, plaque vulnerability, single-cell transcriptomics, spatial transcriptomics

## Abstract

Human atherosclerosis is increasingly recognized as a spatially organized inflammatory disease in which macrophages act as central regulators of lesion evolution rather than as a uniform foam-cell population. Recent single-cell and spatial profiling studies have redefined plaque macrophages as reproducible state programs, including inflammatory, interferon-responsive, lipid-associated, foamy, resident-like, and reparative phenotypes, each embedded within distinct microanatomic niches and multicellular communication networks. These programs are not merely descriptive, but are linked to clinically relevant features such as symptomatic disease, necrotic core expansion, fibrous cap thinning, extracellular matrix remodeling, and recurrent vascular risk. At the same time, the field remains limited by heterogeneity in plaque procurement, anatomic annotation, computational integration, and state nomenclature, which complicates cross-study comparison and obscures biological concordance. This review summarizes the recent advances in human plaque single-cell, spatial transcriptomic, and integrative multi-omics studies to outline the emerging architecture of macrophage states in atherosclerosis. We examine how atlas-scale frameworks connect state definition with spatial localization, regulatory circuitry, and lesion behavior, and discuss how these insights refine mechanistic understanding of plaque progression. We further highlight the translational potential of macrophage-state signatures for risk stratification, molecular imaging, and therapeutic targeting. A coherent human plaque macrophage atlas offers a conceptual and practical framework for moving from descriptive heterogeneity toward clinically actionable biology in atherosclerotic disease.

## 1 Introduction

Human atherosclerosis represents not merely a disorder of lipid deposition, but a spatially orchestrated, multicellular inflammatory process in which macrophages serve as central integrators of microenvironmental cues, derived from modified lipoproteins, apoptotic debris, stromal remodeling, and innate immune surveillance, to determine whether plaques remain clinically quiescent or evolve toward rupture-prone phenotypes (1). Across the atherosclerotic continuum, macrophages do not constitute a homogeneous population, nor can their functional diversity be adequately encapsulated by the conventional M1/M2 dichotomy (2). Instead, recent single-cell and spatial profiling approaches have resolved plaque macrophage heterogeneity into reproducible transcriptional state programs, encompassing inflammatory, interferon-responsive, lipid-associated, foamy, resident-like, and reparative phenotypes, each embedded within discrete microanatomic niches and multicellular communication networks (3–5). Critically, these state programs extend beyond descriptive taxonomy: they exhibit robust associations with clinically salient features, including symptomatic presentation, necrotic core expansion, fibrous cap attenuation, extracellular matrix remodeling, and recurrent vascular risk, thereby establishing a mechanistic link between cellular identity and lesion behavior (6–10).

Nevertheless, progress toward a unified understanding remains constrained by methodological heterogeneity in plaque procurement protocols, anatomical annotation standards, computational integration strategies, and state nomenclature conventions—factors that can obscure biological concordance across independent studies (11–13). A coherent, atlas-scale framework for human plaque macrophages is therefore essential to transcend descriptive cataloguing and establish a unified paradigm that systematically connects state definition, spatial contextualization, regulatory circuitry, and translational applicability. This review summarizes recent advances in single-cell transcriptomics, spatial omics, and integrative multi-omics studies to delineate the emerging architecture of macrophage programming in human atherosclerosis.

## 2 Constructing a human plaque macrophage atlas

### 2.1 Human plaque procurement, anatomic annotation, and clinical phenotyping

The systematic construction of a human plaque macrophage atlas is fundamentally contingent upon procurement protocols that concurrently preserve cellular viability, transcriptional fidelity, and spatial microanatomy. Contemporary carotid studies have therefore leveraged endarterectomy specimens linked to deeply phenotyped clinical cohorts, enabling rigorous correlation of tissue-resident molecular profiles with perioperative symptomatology and longitudinal outcomes (7, 14). Within the AtheroExpress framework, single-cell profiling revealed that macrophage abundance represented the sole major cellular compartment significantly associated with both index symptomatic presentation and three-year secondary cardiovascular events (7), underscoring the centrality of macrophage biology to clinical phenotype. Complementarily, proteomic interrogation of carotid endarterectomy samples identified inflammatory and calcific lesion programs that outperformed conventional imaging and histology in predicting future vascular risk (15). Pre-sequencing methodological variables warrant explicit consideration: cryopreserved human atheroma retain broadly concordant hematopoietic transcriptional profiles relative to fresh tissue, yet exhibit elevated mitochondrial RNA content, indicative of modest stress-associated artifacts necessitating analytical control (16).

Anatomic annotation constitutes a second critical determinant of atlas validity, as intraoperative tissue loss can substantially distort inferred cellular architectures. The absence of LYVE1^+^ macrophages in certain carotid preparations, attributed to adventitial layer loss during endarterectomy, illustrates that sampling geometry directly modulates macrophage-state recovery (16). Spatially resolved approaches mitigate this limitation by enabling compartment-specific mapping of fibrous-cap, core-adjacent, intimal, and medial programs. Such region-resolved analyses have revealed sex-dimorphic patterns: in female fibrous plaques, smooth-muscle-cell-driven extracellular matrix remodeling and endothelial-to-mesenchymal transition localize preferentially to the fibrous cap, whereas male lesions exhibit macrophage-dominant inflammation proximal to the necrotic core (14). Analogously, region-resolved coronary profiling detected instability-associated intimal CD68^+^ subpopulations and distinct calcification-linked signatures distributed differentially across intimal and medial layers (8). Clinical phenotyping must extend beyond luminal stenosis severity to encompass a multidimensional covariate space—including symptom status, biological sex, diabetes mellitus, smoking history, cerebrovascular presentation, plaque composition, and pericarotid lymph node morphology, each of which has stratified inflammatory burden or plaque instability in recent human studies (17–19).

### 2.2 Single-cell and spatial platforms for macrophage state discovery and localization

Single-cell and spatial profiling have fundamentally transformed the resolution of macrophage heterogeneity in atherosclerotic lesions by disentangling transcriptional states, surface phenotypes, and anatomical context, thereby circumventing the limitations of bulk tissue averaging (20–23). In human carotid plaques, integrated scRNA-seq and CITE-seq delineated IL1B-high inflammatory, C1Q-enriched efferocytotic, TREM2-positive foam-cell, and proliferative subsets, with parallel epitope quantification substantially refining annotation confidence beyond transcriptomics alone (20). Scaling this approach to an integrated atlas of 250,000 cells demonstrated that cross-cohort harmonization and surface-protein validation yield portable macrophage taxonomies amenable to automated annotation and bulk-tissue deconvolution (21) Spatial methodologies subsequently restore the microanatomic context essential for functional interpretation. Spatially resolved transcriptomics revealed that VCAM1-driven mitochondrial DNA synthesis and STING-linked inflammatory programs are compartmentalized within plaque macrophages under inflammatory stress in both human and murine lesions (24). In excised carotid specimens, co-registration of near-infrared photoacoustic imaging with spatial transcriptomics and proteomics localized high-risk inflammatory macrophages (CD74^+^/HLA-DR^+^/CD14^+^/CD163^+^) to bilirubin-rich microdomains characteristic of plaque vulnerability (25, 26). Coronary spatial profiling further mapped modulated vascular cells to macrophage-dense neointima, illustrating how cellular neighborhood architecture dictates niche-specific state conditioning (27). Complementary mass cytometry of plaque and peripheral blood revealed CD163^+^ macrophages co-localizing with activated lymphoid subsets, establishing a direct link between tissue-resident states and systemic immune perturbation (28). These multimodal platforms anchor atlas construction by coupling dissociation-based state discovery with spatial validation, thereby redefining macrophage programs as biologically contextualized entities rather than computational artifacts (29–31).

### 2.3 Nomenclature and operational definitions based on gene programs

Computational assembly of a human plaque macrophage atlas demands analytical workflows that suppress technical batch effects while preserving intrinsic lesion biology. Cross-species integration of 12 murine immune scRNA-seq datasets with human coronary and carotid plaques delineated resident (*Lyve1*), inflammatory (*Il1b*), and lipid-loaded (*Trem2*^high^) macrophage subsets, mapping *TREM2*, *SPP1*, *GPNMB*, and *CD9* to a conserved human foamy program and establishing a foundation for reference-guided mapping (32, 33). Human-centric meta-analyses across eight studies subsequently combined scRNA-seq, spatial transcriptomics, and chromatin accessibility data to resolve foamy macrophages into pro-foamy, phagocytic, high-efflux, death-prone, and synthetic substates. Pseudotemporal trajectory inference reconstructed bifurcated differentiation pathways, with orthogonal immunostaining corroborating computational assignments (34, 35). Extending harmonization beyond unsupervised clustering, Mocci et al. anchored single-cell subclusters from asymptomatic and symptomatic carotid plaques to 135 tissue-specific gene-regulatory networks derived from 600 coronary artery disease patients, isolating macrophage GRN33 and GRN122 as clinically associated programs (36, 37).

State nomenclature in human plaque atlases must be grounded in reproducible gene programs rather than reductive M1/M2 dichotomies. CD52^high^ lipid-handling macrophages constitute a discrete operational state, as their transcriptomic profile, chromatin accessibility landscape, and coronary artery disease heritability enrichment converge on a coherent regulatory network centered on FDX1 that mitigates lipoprotein accumulation (38). Similarly, transitional monocyte-to-macrophage intermediates represent a distinct maturation program, operationally defined by elevated chemokine expression and arrested differentiation upon impairment of Srsf3-dependent alternative polyadenylation (39). Lipid-associated macrophages should likewise be classified according to conserved modules anchored by Trem2 and Lpl, retaining tissue-specific auxiliary signatures rather than subsuming them within a monolithic foamy category (40). Crucially, macrophage-like smooth muscle cells in advanced lesions require explicit distinction from lineage-committed macrophages, as their IRF8/NF-κB-driven transcriptional signature reflects phenotypic transdifferentiation rather than myeloid ontogeny (41). Furthermore, state definitions reinforced by METTL3/YTHDF2-mediated epitranscriptomic regulation or CXCL4/Wnt5a/MMP7/S100A8 signaling axes provide robust, functionally grounded frameworks for plaque macrophage classification (42, 43).

## 3 Canonical macrophage state programs in human atherosclerosis

### 3.1 Inflammatory and interferon-responsive programs shaping immune activation

In human atherosclerosis, inflammatory and interferon-responsive macrophage programs are most rigorously defined as coordinated, context-dependent gene modules rather than as binary M1/M2 states. Within contemporary human plaque atlases, inflammatory macrophages are characterized by elevated expression of IL1B, TNF, NF-κB-target genes, and chemokines that promote leukocyte recruitment; their abundance consistently correlates with symptomatic disease presentation and histological features of plaque instability (6). Spatially resolved analyses further reveal that these programs are not uniformly distributed but instead concentrate at anatomically defined interfaces (the fibrous cap, shoulder region, and necrotic core boundary) where oxidized lipids, hypoxic stress, and cellular debris generate persistent danger-associated molecular patterns (44). Notably, macrophage populations separated by mere millimeters within these microenvironments can adopt divergent effector states, underscoring the fine-grained spatial regulation of macrophage functional heterogeneity. To mitigate semantic inconsistency across independent studies, harmonized ontological frameworks partition the inflammatory axis into partially dissociable subprograms: IL-1β^high^, complement-enriched, and antigen-presentation-dominant modules. Such program-level definitions confer greater portability and mechanistic interpretability than morphology-based or marker-limited classifications (21). Critically, inflammatory states frequently occupy a transcriptional continuum with lipid-associated phenotypes (6): under conditions of prolonged lipid loading, impaired efferocytosis, or chronic oxidative stress, lipid-associated macrophage populations, including TREM2^+^ subsets, can transition from adaptive lipid handling toward maladaptive, cytokine-producing states (45).

Interferon-responsive programs form a second major axis of macrophage heterogeneity. These states are marked by STAT/IRF-centered circuits, ISGs, and in some contexts an Antimicrobial-like Stress Response that coexists with or partially substitutes for canonical NF-κB inflammation (46). Chromatin-accessibility data support the regulatory basis of this program by identifying IRF, STAT, and inflammatory motif activity in plaque macrophage states (47). Several innate-sensing pathways connect this interferon wiring to plaque inflammation. Mitochondrial stress and GSDMD activity promote mtDNA release into the Cytosolic compartment, activating CGAS-STING, RF3, and NF-KB signaling and thereby integrating interferon and inflammatory transcriptional outputs (48, 49). Aberrant mitochondrial DNA accumulation provides a related mechanism that amplifies macrophage activation and helps explain the repeated appearance of mtDNA- and interferon-linked signatures in plaque atlases (24). Inflammasome pathways intersect with both axes through feed-forward loops linking innate sensing, lytic death, and cytokine release. AIM2 activation can intensify inflammatory output, while IL-1B-centered amplification further reinforces local macrophage activation (50). Besides, GSDME-dependent pyroptosis promotes inflammation and lesion progression by increasing DAMPs, thereby sustaining NF-κB and interferon-sensing circuits and contributing to necrotic core expansion (51, 52). Counter-regulatory metabolism can restrain these programs: the IRG1-itaconate pathway buffers cholesterol-driven inflammation and shifts macrophages away from excessive IL1B production (53).

**Figure 1:**
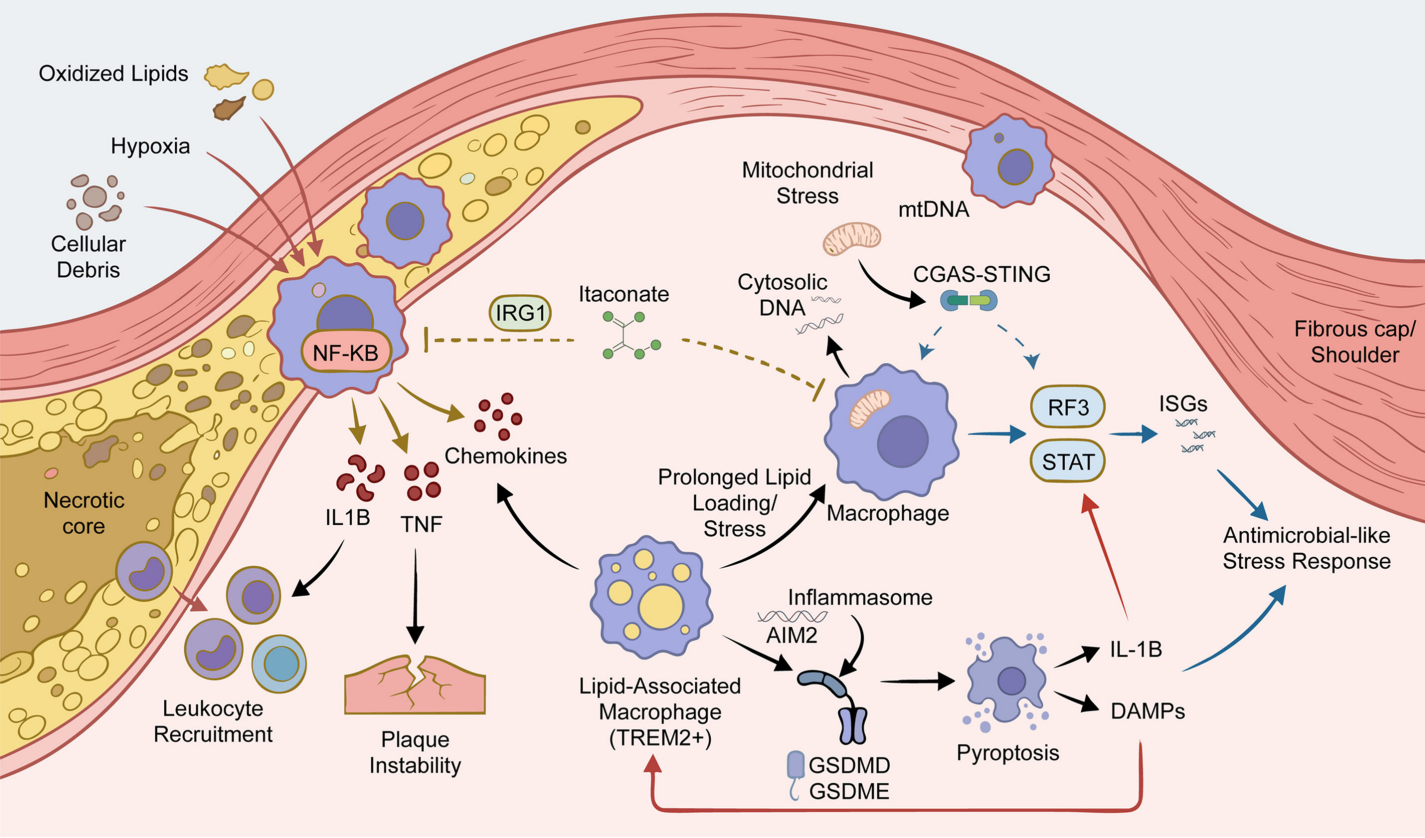
Inflammatory and interferon-responsive macrophage programs in atherosclerosis. The diagram maps these programs within the fibrous cap/shoulder and necrotic core interface, where oxidized lipids, hypoxia, and cellular debris promote NF-κB-driven cytokine and chemokine expression, leukocyte recruitment, and plaque instability. Prolonged lipid loading supports TREM2+ lipid-associated macrophages, whereas mitochondrial stress, cytosolic DNA, and cGAS–STING signaling induce STAT/IRF3-dependent interferon-stimulated genes. Inflammasome activation and GSDMD/GSDME-mediated pyroptosis amplify IL-1β and DAMP release.

Spatial transcriptomic analyses further reveal that plaque regions associated with stability versus instability exhibit fundamentally distinct immune-activation architectures, thereby anchoring interferon-responsive and inflammatory macrophage programs to clinically pertinent microenvironments rather than to artifacts of tissue dissociation (54). These spatial heterogeneities frequently co-localize with microdomains enriched for IFN-γ, MHC molecules, and chemokine signaling, indicating that niche-specific cues actively sculpt macrophage state occupancy rather than merely reflecting passive cellular accrual. This two-axis framework implies that therapeutic inhibition of IL-1β/NF-κB signaling alone may prove insufficient to resolve interferon-high macrophage states; by contrast, targeting mitochondrial stress, cytosolic DNA sensing, or the cGAS–STING pathway may preferentially attenuate high-risk niche programs linked to plaque vulnerability (55). Integrated atlas-level interpretation therefore requires explicit program-based definitions that remain stable across preprocessing pipelines, cross-cohort mapping, orthogonal validation, and anatomic context. This pattern is especially evident in human carotid and coronary plaques, where unstable lesions show stronger inflammatory-interferon coupling across neighboring niches (**Figure 1**).

### 3.2 Lipid-associated and foamy programs coupling lipid handling with oxidative stress

In human atherosclerosis, lipid-associated and foamy macrophage programs transcend passive lipid-storage phenotypes to function as integrated transcriptional and metabolic modules in which oxidized LDL uptake, impaired cholesterol efflux, and oxidative stress are tightly coupled. Single-cell profiling of carotid plaques has delineated a pathogenic trajectory wherein lipid-associated macrophages evolve into a PLIN2-high/TREM1-high inflammatory subset that correlates with cerebrovascular events, demonstrating that lipid loading can potentiate, rather than suppress, inflammatory output (6). This maladaptive shift is reinforced by signaling circuits that augment lipid acquisition: Gαs enrichment in cholesterol-metabolizing macrophages drives cAMP–CREB-dependent upregulation of CD36 and SR-A1, thereby accelerating oxLDL internalization and foam-cell expansion (56). Concurrently, counter-regulatory efflux and clearance pathways are frequently compromised. For instance, Listerin-mediated K63-linked ubiquitination normally stabilizes ABCA1 to sustain cholesterol efflux; its depletion precipitates accelerated lipid deposition and necrotic core formation (57). Similarly, ASIC1–RIP1 signaling impedes TFEB-dependent lipophagy, promoting intracellular lipid retention and metabolic stress (58).

Additional lesion-amplifying mechanisms further destabilize the lipid-handling equilibrium. TRIM31 deficiency stabilizes LOX-1, exacerbating macrophage lipid uptake and inflammatory signaling (59), while CTSB-mediated degradation of ferroportin disrupts iron homeostasis and triggers ferroptosis-associated stress (60). TREM2-associated pyroptotic programs also emerge as key drivers of plaque instability (61). Beyond structural accumulation, specific lipid species function as active instructive signals: lipoprotein(a)-enriched diacylglycerols directly provoke IL-6, IL-8, and IL-1β production, activate NLRP3-dependent inflammasome signaling, and enhance transendothelial migration (62). At the tissue level, vulnerable plaques exhibit marked enrichment of glycolytic enzymes, including HK3, PKM, and LDHA, defining a metabolically strained foamy niche wherein lipid processing, ROS burden, and inflammatory amplification converge (63, 64). Reanalysis of human plaque transcriptomes further identified ENO1 enrichment within CD68^+^ inflammatory macrophages; disruption of the ApoB100–ENO1 interaction attenuated oxLDL uptake, cytokine release, and foam-cell formation, positioning modified lipoprotein sensing as a critical amplifier of lipid-stressed macrophage states (65). In contrast, resident-like and reparative macrophage programs in atherosclerosis are characterized by homeostatic scavenging, extracellular matrix support, and active suppression of destructive inflammation rather than maximal cytokine production (66, 67). CD163 demarcates a tissue-resident macrophage compartment; CD163-targeted molecular imaging has confirmed its enrichment in advanced human plaques, underscoring its stability and potential as a translational biomarker. A reparative trajectory is further evidenced by macrophages following anti-miR-33 therapy, which upregulate matrix-stabilizing genes (Col1a2, Col2a1, Col3a1, Fn1, Timp3) while repressing Mmp12 during plaque regression, consistent with fibrotic stabilization and tissue repair (68). Conversely, LYVE1^+^/CCL24^+^ resident-like subsets can paradoxically drive osteogenic reprogramming of vascular smooth muscle cells and promote calcification.

## 4 Spatial niches and multicellular circuits that condition macrophage states

### 4.1 Microanatomic localization across cap, shoulder, core, and adventitia

Spatially resolved profiling reveals that plaque macrophage states are not randomly dispersed but instead exhibit precise microanatomic organization, with distinct transcriptional programs segregated across defined lesion compartments. In carotid atherosclerosis, inflammatory HMOX1^+^ macrophages preferentially localize to subluminal regions and border zones spanning the fibrous cap and necrotic core, where they appear to undergo local transition toward lipid-handling TREM2^+^ states; concurrently, macrophage-dense hotspots map to cellular neighborhoods enriched for neovascularization and histological features of lesion vulnerability (9). Fibrous cap architecture is further shaped by ACTA2^+^ myofibroblast-like cells of heterogeneous origin—including populations arising via macrophage-to-mesenchymal transition—thereby linking cap integrity to multicellular remodeling dynamics rather than to a canonical smooth-muscle compartment alone (69). In advanced fibroatheroma, lumican-expressing fibroblast-like cells accumulate preferentially at the necrotic core interface, whereas adventitial tertiary lymphoid organ-like structures harbor expanded B-cell niches whose IgG-secreting activity may potentiate macrophage activation and elevate cerebrovascular risk (70, 71).

### 4.2 Cell–cell communication networks in tune plaque stability

Within human atherosclerotic plaques, macrophage state occupancy is actively tuned by multicellular communication networks rather than by isolated lipid loading alone. In symptomatic carotid plaques, spatial transcriptomics demonstrates that elevated intraplaque LIGHT/TNFSF14 correlates with expanded necrotic cores, heightened inflammatory cytokine burden, and upregulated expression of fibromodulin alongside matrix metalloproteinases (MMP1, MMP2, MMP9, and MMP10), directly coupling immune activation to extracellular matrix turnover in rupture-prone microdomains (72). A complementary coronary proteomic analysis across AHA stages from adaptive intimal thickening to ruptured plaque further showed progressive dysregulation of complement-coagulation pathways in macrophages and fibroblasts, while matrix-organizing programs were concentrated in fibroblasts and smooth muscle cells, supporting a coordinated plaque-destabilizing network rather than a single-cell defect (73). CD163^+^ macrophages from hemorrhagic plaques induce endothelial-to-mesenchymal transition (ETM) via cytokine-activated NF-κB–Snail signaling, downregulating VE-cadherin and CD31 while upregulating transgelin and FSP1 (74). In parallel, macrophage-derived TIMP1 engages CD74 to activate AKT and ERK1/2 in monocytes and promote vascular smooth muscle cell proliferation, establishing a secondary paracrine axis that amplifies inflammatory cell recruitment and accelerates cap remodeling (75). Fibrotic crosstalk further exhibits spatial compartmentalization: TREM2-high and SPP1^+^ macrophage subsets engage fibroblast-like targets through osteopontin-dependent programs, thereby augmenting collagen deposition and driving the expansion of profibrotic progenitors within vascular and perivascular niches (76, 77). Endothelial dysfunction extends beyond a passive upstream trigger: endothelial-derived CSF1 orchestrates monocyte recruitment and inflammatory signaling to expand tissue-degrading macrophage programs, whereas disturbed-flow-induced endothelial reprogramming generates immune-like and foam-like endothelial phenotypes that reciprocally reinforce macrophage-rich unstable microenvironments (78, 79). Consistently, injury mapping showed macrophages communicate with VCAM1^+^ proinflammatory smooth muscle cells, and coculture experiments demonstrated stronger macrophage chemotaxis toward that phenotype, underscoring local feed-forward loops between myeloid cells and remodeling mural cells (80).

## 5 Translational opportunities enabled by atlas-scale signatures

Atlas-scale macrophage signatures now enable clinically actionable translation across biomarker discovery, molecular imaging, and mechanism-guided intervention. Cross-tissue single-cell and TRAP-seq profiling identified conserved macrophage programs between murine and human plaques, nominating soluble TREM2 as a circulating marker distinguishing asymptomatic from symptomatic disease, while plaque-enriched FOLR2 and SLC7A7 emerged as PET-traceable targets; notably, *Slc7a7* loss attenuated acetylated LDL uptake and suppressed lipid-associated programs, directly linking atlas-defined markers to foam-cell biology (81). State-informed molecular imaging further validates this approach: SST2 PET/MRI localized signal to plaque macrophages with high diagnostic performance, exhibiting arterial tissue-to-blood ratios 34.6% higher than post-infarction baselines and treatment-associated signal reduction during follow-up, confirming that macrophage-resolved targets dynamically report inflammatory burden *in vivo* (82). Moreover, ultra-selective carbon nanotubes taken up almost exclusively by Ly-6Chi monocytes and foamy macrophages generated approximately six-fold stronger photoacoustic plaque signal than controls, illustrating how immune-state specificity can improve non-invasive detection of inflamed lesions (83). Genetic anchoring also sharpens target prioritization, as CAD-risk alleles at 15q26.1 reduced FES expression, and Fes deficiency increased plaque size together with monocyte/macrophage accumulation, nominating restoration of FES activity as atheroprotective (84). Beyond canonical myeloid compartments, atlas-guided plasticity maps identify interventional nodes in smooth-muscle-derived macrophage-like states: ChemR23 constrains synthetic switching and remodels cholesterol flux (85); IRF7 knockdown reduces necrotic core formation while improving cap stability (86); ADAMTS7 drives AP-1/PU.1/CD36-dependent smooth muscle foam-cell expansion (87); and SMC-specific Cd47 deletion attenuates macrophage accumulation and necrotic area (88).

## 6 Conclusion

Human atherosclerosis is now recognized not as a passive lipid-storage disorder, but as a spatially orchestrated inflammatory process wherein macrophages function as central integrators of metabolic stress, innate immune sensing, stromal remodeling, and tissue injury. Plaque macrophages do not constitute a homogeneous compartment, nor can their functional diversity be encapsulated by the conventional M1/M2 dichotomy. Instead, single-cell and spatial profiling resolve this heterogeneity into reproducible state programs—inflammatory, interferon-responsive, lipid-associated, foamy, resident-like, and reparative—each confined to discrete microanatomic niches and governed by distinct intercellular circuits. These transcriptional states are clinically consequential, directly correlating with necrotic core expansion, fibrous cap attenuation, matrix degradation, symptomatic presentation, and recurrent vascular risk. However, field-wide progress remains constrained by heterogeneity in tissue procurement, anatomical annotation, computational integration, and state nomenclature. Establishing a standardized, atlas-scale framework is therefore essential to harmonize state definition, spatial context, regulatory circuitry, and translational application, ultimately advancing precision diagnostics and targeted therapeutics in atherosclerotic disease.
